# Sex differences in myocardial remodeling and extracellular volume in aortic regurgitation

**DOI:** 10.1038/s41598-023-37444-y

**Published:** 2023-07-13

**Authors:** Maan Malahfji, Alpana Senapati, Dany Debs, Mujtaba Saeed, Bhupendar Tayal, Duc T. Nguyen, Edward A. Graviss, Dipan J. Shah

**Affiliations:** 1 Cardiovascular MRI Laboratory, Division of Cardiovascular Imaging, Houston Methodist DeBakey Heart & Vascular Center, 6550 Fannin Street, Smith Tower - Suite 1801, Houston, TX 77030 USA; 2 Department of Pathology and Genomic Medicine, Houston Methodist Hospital Research Institute, Houston, TX USA

**Keywords:** Cardiology, Valvular disease

## Abstract

Whether sex differences exist in the cardiac remodeling related to aortic regurgitation (AR) is unclear. Cardiac magnetic resonance (CMR) is the current non-invasive reference standard for cardiac remodeling assessment and can evaluate tissue characteristics. This prospective cohort included patients with AR undergoing CMR between 2011 and 2020. We excluded patients with confounding causes of remodeling. We quantified left ventricular (LV) volume, mass, AR severity, replacement fibrosis by late Gadolinium enhancement (LGE), and extracellular expansion by extracellular volume fraction (ECV). We studied 280 patients (109 women), median age 59.5 (47.2, 68.6) years (*P* for age = 0.25 between sexes). Women had smaller absolute LV volume and mass than men across the spectrum of regurgitation volume (RVol) (*P* ≤ 0.01). In patients with ≥ moderate AR and with adjustment for body surface area, indexed LV end-diastolic volume and mass were not significantly different between sexes (all *P* > 0.5) but men had larger indexed LV end systolic volume and lower LV ejection fraction (*P* ≥ 0.01). Women were more likely to have NYHA class II or greater symptoms than men but underwent surgery at a similar rate. Prevalence and extent of LGE was not significantly different between sexes or across RVol. Increasing RVol was independently associated with increasing ECV in women, but not in men (adjusted *P* for interaction = 0.03). In conclusion, women had lower LV volumes and mass than men across AR severity  but their ECV increased with higher regurgitant volume, while ECV did not change in men. Indexing to body surface area did not fully correct for the cardiac remodeling differences between men and women. Women were more likely to have symptoms but underwent surgery at a similar rate to men. Further research is needed to determine if differences in ECV would translate to differences in the course of AR and outcomes.

## Introduction

Aortic regurgitation (AR) is a common form of valvular heart disease (VHD) characterized by volume and pressure overload of the left ventricle (LV)^1–3^. The natural history of AR has been characterized by progressive LV dilatation and hypertrophy in response to the hemodynamic load on the ventricle, with increased wall stress and eventual manifest LV dysfunction^4–6^. Myocardial remodeling varies significantly among individuals with different forms of VHD, and includes adaptive and maladaptive changes in ventricular shape, volume, cellular, and extracellular components^7^.

Cardiac magnetic resonance (CMR) is an accurate and reproducible non-invasive method to assess ventricular volumes, mass, and tissue characteristics. In addition to a direct and accurate assessment of aortic regurgitant volume (RVol), CMR can assess myocardial replacement fibrosis (with the late gadolinium enhancement technique, [LGE]) and interstitial expansion (by calculating extracellular volume fraction [ECV] from T1 mapping before and after gadolinium contrast administration). Myocardial fibrosis in AR has been described in histopathology studies and was characterized by increased fibronectin and non-collagen components^8^. In a recent study, ECV measured by CMR was strongly correlated with interstitial fibrosis measured on histology at the time of surgery in patients with severe AR^9^.

Important sex differences in myocardial remodeling have been described in aortic stenosis (AS)^10–12^ and primary mitral regurgitation^13^. However, there is less clarity on the sex differences in the adaptive response to AR^14^ and particularly fibrosis burden. The aim of this study was to assess if there are sex differences in myocardial remodeling and fibrosis in patients with isolated AR.

## Methods

### Patient selection

Patients undergoing a contrast CMR at Houston Methodist Hospital (Houston, Texas) were enrolled into a prospective observational CMR registry (DEBAKEY-CMR registry, NCT04281823). We included patients with AR enrolled between July 2011 and March 2020. The determination of AR presence and its severity was done according to the CMR results. No patients with acute AR (i.e. aortic dissection, acute endocarditis) were included. Patients enrolled in the registry undergo a thorough baseline patient interview and review of medical records at the time of imaging. Patients were excluded if they had any confounding causes of LV fibrosis by *clinical history*: (1) obstructive coronary artery disease (CAD), history of myocardial infarction, or coronary revascularization; (2) cardiomyopathy deemed unrelated to AR (i.e., amyloidosis or sarcoidosis); or (3) prior cardiac surgery or transcatheter structural intervention. To avoid ambiguity regarding confounding etiologies of any LV fibrosis detected, we further excluded patients with the following findings on CMR: congenital heart disease and aortic coarctation, coexisting other valvular disease including AS that was greater than mild in severity (based on integrating valve area and peak velocity measured on CMR), except secondary tricuspid regurgitation deemed related to AR. We also excluded patients with cardiac devices where susceptibility artifacts limit accurate T1 mapping^15–19^.

**Figure 1:**
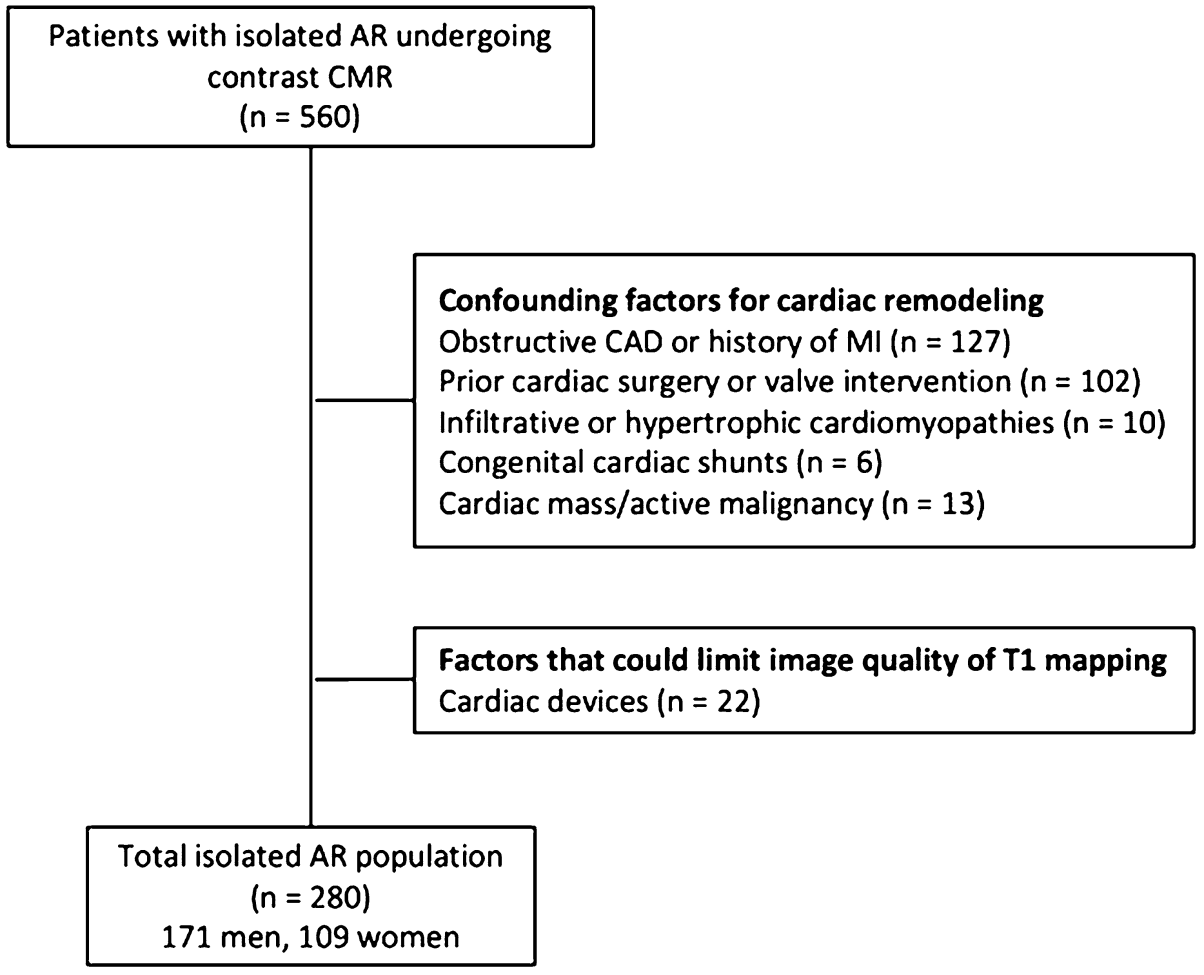
Detailed description of patient enrollment. *AR* aortic regurgitation, *CAD* coronary artery disease, *CMR* Cardiac magnetic resonance, *MI* myocardial infarction.

The patient enrollment process is summarized in Fig. 1. The study was approved by the institutional review board at Houston Methodist Research Institute, and patients gave written informed consent. The study was conducted in accordance with the principles outlined in the Declaration of Helsinki.

### CMR study protocol

CMR images were acquired using either 1.5- or 3.0-T clinical scanners (Siemens Avanto, Aera, Verio, and Skyra, Siemens, Erlangen, Germany) with phased-array coil systems. CMR examinations began with cine-CMR for anatomic and functional assessment in a short-axis stack, and standard 2-, 3-, and 4-chamber views using a steady-state free-precession (SSFP) sequence with typical flip angle of 65° to 85°; repetition time (TR) of 3.0 ms; echo time (TE) of 1.3 ms; in-plane spatial resolution of 1.7 to 2.0 mm × 1.4 to 1.6 mm; slice thickness of 6 mm, with 4 mm interslice gap; and temporal resolution of 35–40 ms. Anatomic assessment of the aortic valve was performed with the use of cine SSFP sequences. The 3-chamber view and coronal LV outflow views were used to prescribe a parallel series of at least 3 thin (4–5 mm) slices in short axis to provide assessment of the aortic valve’s morphology. Phase contrast CMR was performed at the level of the ascending aorta, sinotubular junction, left ventricular outflow tract (LVOT) and the pulmonary artery. The typical parameters were flip angle of 25°–30°, TR ~ 5 ms, TE of 2.4 ms, reconstructed in-plane spatial resolution of ~ 2.0 × 2.4 mm, slice thickness of 6 mm, and temporal resolution of ~ 40–50 ms. The initial velocity encoding used was 150 cm/s for at the LVOT and pulmonary artery, and 250 cm/s at the sinotubular junction. Adjustments were made according to the peak velocity across the aortic valve^15–19^.

LGE imaging was performed using a magnitude and phase-sensitive segmented inversion-recovery sequence, approximately 10 min after intravenous gadolinium contrast administration (gadopentetate dimeglumine or gadoterate meglumine, 0.15 mmol/kg). Parameters were in-plane spatial resolution of 1.8 × 1.3 mm and slice thickness of 6 mm, with inversion time adjusted to null normal viable myocardium. Cine- and LGE-CMR images were obtained in matching short- and long-axis planes to cover the entire ventricle^16–18,20^.

The ECG triggered modified Look Locker inversion recovery (MOLLI) sequence was used for assessment of myocardial T1 relaxation times in a mid-ventricular slice in all patients. Native myocardial T1 relaxation time was measured before administration of contrast. Acquisition scans for post contrast T1 mapping were performed after the standard delayed enhancement acquisition protocol for a mid- ventricular matching slice, approximately 15–20 min after the infusion of the contrast agent. Parameters for MOLLI technique include slice thickness of 6 mm, voxel size 2.1 × 1.6 × 6 mm, TE = 1.09, TR = 675, flip angle = 35°, twofold parallel imaging. Precontrast 5(3)3 and post-contrast 4(1)3(1)2 heart beat sampling schemes were used on the 1.5-Tesla scanner and pre-contrast 5(4)2 and post-contrast 4(1)2(2)2 heart beat sampling schemes on the 3.0-Tesla scanner. Shimming and delta frequency adjustments were applied to minimize off-resonance artifacts^15,16,18,20^.

### CMR analysis

Ventricular volumes were measured by planimetry of the endocardial borders, on a stack of short-axis images acquired from cine-CMR covering both ventricles from base to apex. Papillary muscles and trabeculae were excluded from blood volume. LV end-diastolic volume (EDV), LV end-systolic volume (ESV), right ventricular (RV) EDV, and RV ESV were calculated by summation of these images. The stroke volume for each ventricle was determined by subtracting the ESV from the EDV. The LVEF and RVEF were calculated by dividing the stroke volume by the EDV of the respective ventricle. LV mass was calculated by tracing LV epicardial and endocardial borders at end diastole and assuming myocardial density of 1.05 g/ml. Papillary muscles and trabeculations were included in LV mass. Left ventricular dilatation and hypertrophy were defined as an indexed LVEDV and LV mass > 95th percentile of the normal range, according to age and sex^21^.

The presence and extent of LGE was assessed in all LV segments according to the American College of Cardiology/American Heart Association 17-myocardial-segment model by a consensus of 2 readers who were blinded to clinical history and other imaging information. To mitigate the effect of imaging artifacts, replacement fibrosis was only considered present if it was visually identified on 2 contiguous or orthogonal slices and seen on both magnitude and phase-sensitive image reconstruction^22^.

We used the full width half maximum method to quantify the extent of LGE. The RVol was calculated using the direct method from phase contrast imaging at the level of the sinotubular junction or via the difference between LV outflow tract forward flow and net pulmonary artery flow. The regurgitant fraction was calculated as (reverse volume/forward volume * 100%). Analysis of LV volume, mass, AR severity, and LGE extent was performed on Precession (Heart Imaging Technologies).

Analysis of T1 values was performed using manually contoured regions of interest, for both pre and post contrast midventricular slices. Extracellular volume (ECV) was calculated as: ECV = ∆R1 myocardium/∆R1 blood × (100 − hematocrit), where ∆R1 myocardium or change in relaxation rate is given by: 1/T1 myocardium post contrast injection − 1/T1 myocardium pre-contrast injection, and ΔR1 blood = 1/T1 blood-post contrast − 1/T1 blood-pre-contrast administration. Hematocrit was measured on a venous blood sample obtained at the time of CMR. By this technique an estimate of T1 is encoded in the intensity of each pixel. ECV, T1 pre-contrast and T1 post contrast values were calculated separately for each of the six segments in the midventricular slice before and after contrast administration. We applied the ECV analysis to the six segments at the mid-ventricular level. Measurements were calculated as an average of all segments after censoring segments with LGE or artifact (only 6 segments had artifacts). An offset of 20% from epicardial and endocardial contours was applied to the T1 sequences to reduce bias from partial volume effects.

CMR42 Version 5.6 (Circle Cardiovascular, Calgary, Canada) was used for ECV analysis^16,18,19,23,24^.

### Follow up

Clinical follow-up was initiated from the time of CMR imaging. Event data and last follow up date were gathered from medical record review, telephone interviews with the patients, relatives, or their health care providers. Management plans including surgery vs medical therapy was ascertained.

### Statistical analysis

Distribution of continuous variables was tested by The Kolmogorov–Smirnov test. Data was presented as frequencies and percentages for categorical variables and as mean (standardized deviation, SD) or median (25th, 75th percentile) for continuous variables. Comparisons between groups were conducted using the Student’s t tests or Mann Whitney U tests for continuous variables and chi-square or Fisher exact tests for categorical variables as appropriate. The differences in ventricular volume, mass, and ejection fraction between men and women according to RVol were evaluated by the generalized linear models (GLM). Subgroup analysis was done in patients with RVol > 30 ml. Factors associated with ECV fraction were determined by the multivariable GLM. Variable selection for the multivariable model was conducted based on the clinical importance and also using the Stata’s Lasso technique with the cross-validation selection option. The interaction between sex and RVol in the association with ECV fraction was evaluated using the spline interpolation and also in the GLM model. The analyses were performed on SPSS version 21 (IBM SPSS Statistics, IBM Corporation, Armonk, New York) and Stata version 16.1 (StataCorp LLC, College Station, TX, USA). A *P* value of < 0.05 was considered statistically significant.

## Results

### Study population

Patient characteristics are presented in Table 1. The study population had a median age of 59.5 (47.2, 68.6) years and included 171 men and 109 women. No significant difference in age was noted between men and women, *P* = 0.25. The prevalence of hypertension was similar between sexes (57.3% in men and 54.1% in women, *P* = 0.60) as was the systolic blood pressure (*P* = 0.84). Women had a slightly lower diastolic blood pressure than men (*P* = 0.01). Men and women had a similar prevalence of diabetes (6% of men vs 6.4% of women, *P* = 0.84) and hyperlipidemia (46.8% of men and 40.4% of women, *P* = 0.29). There were 24/280 (8.5%) of patients with mild AS and the remainder had no AS. No significant difference in the prevalence of AS was found between men and women (*P* = 0.66).

**Table 1:** Baseline clinical findings.

Characteristic	Total	Men	Women	*P* value
Age (years)	59.5 (47.2, 68.6)	58 (46.9, 68.1)	61.4 (48.1, 69.9)	0.25
Caucasian (%)	209 (74.6)	125 (73.1)	84 (77.1)	0.78
BSA (m^2^)	2.0 (1.79, 2.1)	2.0 (1.98, 2.22)	1.73 (1.6, 1.9)	< 0.001
BMI (Kg/m^2^)	26.6 (23.7, 30.5)	27.3 (24.4, 31.3)	25.1 (21.7, 29.4)	< 0.001
SBP (mmHg)	132 (121, 143)	132 (124, 143)	132 (118, 142)	0.42
DBP (mmHg)	71 (64, 80)	73 (65, 81)	69 (62, 78)	0.01
Clinical characteristics				
Hypertension (%)	157 (56.1)	98 (57.3)	59 (54.1)	0.60
Diabetes (%)	19 (6.8)	12 (7)	7 (6.4)	0.84
Hyperlipidemia (%)	124 (44.3)	80 (46.8)	44 (40.4)	0.29
Atrial fibrillation/flutter*	21 (7.5)	10 (5.8)	11 (10.1)	0.18
Active smoking (%)	70 (25.4)	47 (27.8)	23 (21.5)	0.18
Medications				
Beta blockers (%)	106 (37.9)	66 (38.6)	40 (36.7)	0.74
ACE inhibitor (%)	63 (22.5)	43 (25.1)	20 (18.3)	0.18
ARB (%)	57 (20.4)	41 (24)	16 (14.7)	0.06
Spironolactone (%)	7 (2.5)	6 (3.5)	1 (0.9)	0.17
Diuretics (%)	63 (22.5)	37 (21.6)	26 (23.9)	0.66

Values are mean ± SD, n (%), or median (interquartile range).

*BMI* body mass index, *BSA* body surface area, *ACE* angiotensin converting enzyme, *ARB* angiotensin receptor blocker, *DBP* diastolic blood pressure, *SBP* systolic blood pressure.

The *P* values are results for the t-test, Mann Whitney U test, or the chi-square test.

*Atrial fibrillation or flutter during the scan.

### Sex differences in myocardial cavity remodeling

**Figure 2:**
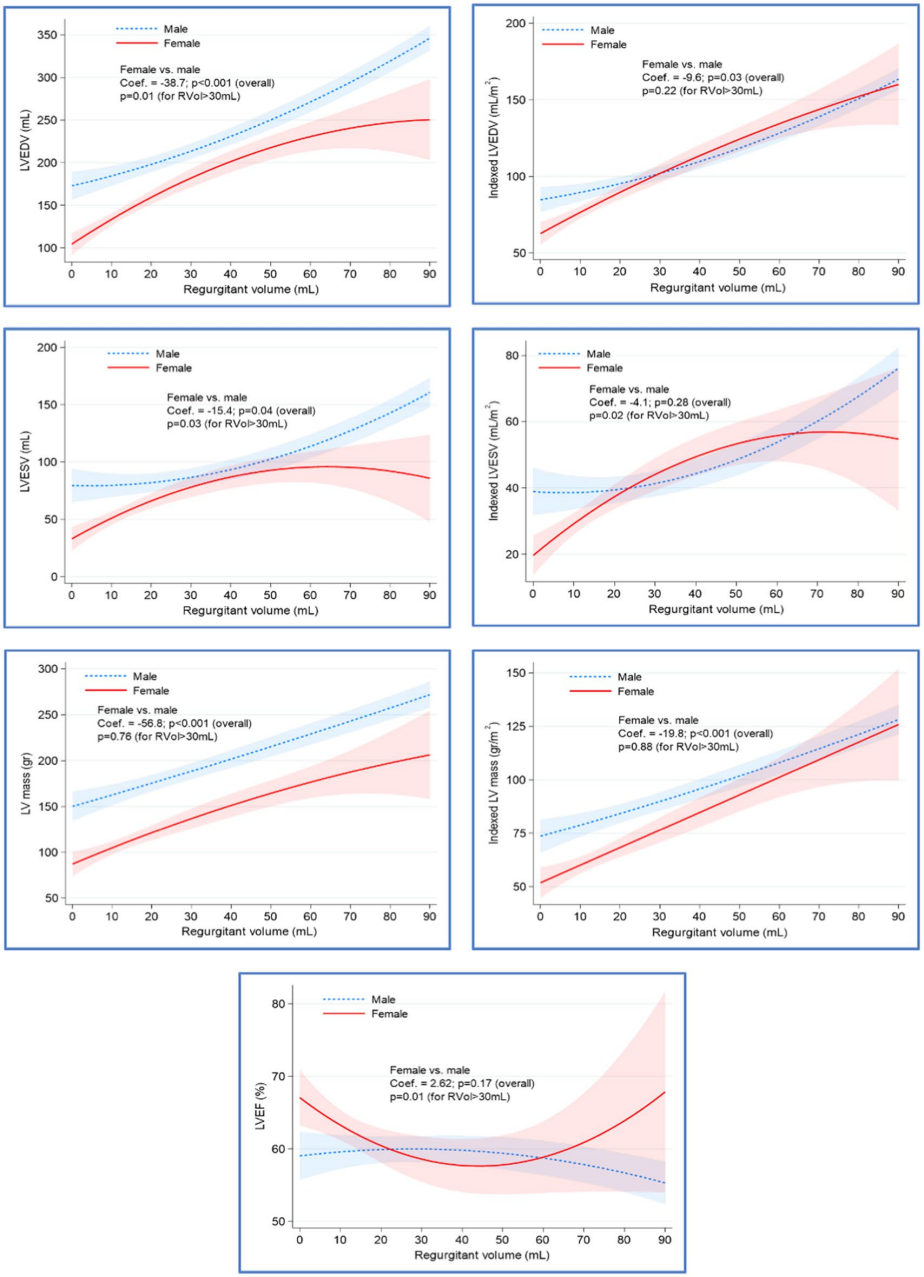
Sex differences in cardiac remodeling across degrees of AR severity. The association between the regurgitant volume (RVol) and absolute/ indexed ventricular volumes, mass and LVEF variables were depicted by the quadratic prediction plots with confidence interval bands and stratified by gender. The coefficients representing the difference in the evaluated variables between genders were obtained from the multivariable generalized linear modeling (GLM) in all patients. The *P*-values representing the difference in the evaluated variables between genders were obtained from the similar GLM models in patients having aortic RVol > 30 mL. The GLM models were adjusted for gender, diabetes, RVol, aortic valve morphology, and interaction term of aortic Rvol and sex. *LVEDV* left ventricular end diastolic volume, *LVESV* left ventricular end systolic volume, *LVEF* left ventricular ejection fraction.

Findings on CMR are presented in Table 2. Men were more likely to have a bicuspid aortic valve (42.7% of men vs. 26.6% of women, *P* = 0.006). Women had smaller absolute LVEDV, LVESV, and mass across RVol degrees (*P* ≤ 0.04). Adjustment to body surface area showed an overall higher indexed LVEDV and indexed LV mass in men primarily in lower degrees of regurgitant volume. In ≥ moderate AR (RVol > 30 ml), no significant differences in indexed LVEDV and indexed LV mass between men and women were noted, but indexed LVESV was higher in men along with a lower LVEF. There was no significant interaction with sex and RVol in the association with LVEF (Fig. 2).

**Table 2:** CMR findings.

Characteristic	Total	Men	Women	*P* value
Bicuspid aortic valve (%)	102 (36.4)	73 (42.7)	29 (26.6)	0.006
Mild aortic stenosis	24 (8.6)	16 (9.4)	8 (7.3)	0.66
Indexed LVEDV (ml/m^2^)	95.5 (77.1, 120.3)	107.7 (84.0, 136.2)	81.2 (67.8, 98.6)	< 0.001
Indexed LVESV (ml/m^2^)	37.0 (27.0, 50.6)	41.3 (31.2, 64.0)	30.1 (21.7, 43)	< 0.001
LVEF (%)	61 (55, 66.4)	60.6 (55, 65.1)	62 (56.8, 69.4)	0.01
Indexed LV mass (gr/m^2^)	78.8 (61.3, 101.3)	88.6 (72.9, 114.4)	60.5 (51.5, 85.2)	< 0.001
Indexed RVEDV (ml/m^2^)	80.8 (69.6, 98.5)	86.6 (74.1, 106.6)	74 (65.3, 85.2)	< 0.001
Indexed RVESV (ml/m^2^)	37.5 (29.8, 45.7)	40.2 (33.0, 49.7)	31.6 (24.1, 38.6)	< 0.001
RVEF (%)	55 (50, 59.9)	53 (48.5)	57 (53, 63)	< 0.001
LA Volume index (ml/m^2^)	47.9 (39.2, 59.4)	48.2 (38, 59.5)	47.6 (40.1, 59.7)	0.94
Aortic RVol				
< 15 ml	108 (38.6)	48 (28.1)	60 (55)	< 0.001
15–30 ml	68 (24.3)	39 (22.8)	29 (26.6)	
> 30 ml	104 (37.1)	84 (49.1)	20 (18.3)	
LGE burden (grams)	4.4 (3.2)	4.9 (4.3)	3.6 (2.3)	0.07
ECV fraction (%)	25.5 (23.7, 28)	25.2 (23,0, 27.6)	26.4 (24.0, 28.6)	0.03
ECV in RVol < 15 ml	25.5 (23.8, 28.1)	25.3 (24.0, 28.3)	25.6 (23.1, 27.9)	0.85
ECV in RVol 15–30 ml	25.3 (23.7, 27.2)	24.5 (22.3, 26.0)	26.8 (24.0, 28.5)	0.002
ECV in RVol > 30 ml	25.8 (23.7, 28.5)	25.4 (23.1, 28.0)	28.0 (24.9, 29.2)	0.03

Values are mean ± SD, n (%), or median (interquartile range).

*LVEDV* left ventricular end diastolic volume, *LVESV* left ventricular end systolic volume, *LVEF* left ventricular ejection fraction, *LV* left ventricular, *RVEDV* right ventricular end diastolic volume, *RVESV* right ventricular end systolic volume, *RVEF* right ventricular ejection fraction.

The *P* values are results for the t-test, Mann Whitney U test, or the chi-square test.

### Sex differences in myocardial replacement fibrosis

LGE was present in 46 (16.4%) of patients and the prevalence was similar in men (18.7%) vs women (12.8%), *P* = 0.19. The median (interquartile range) LGE burden was 2.55 (1.77, 4.20)% of the myocardium, which was not significantly different between men (2.4 [1.72, 3.50])% and women (3.2 [1.77, 5.0])%, *P* = 0.22. No statistically significant association was present between LGE burden and ECV (r = − 0.07, *P* = 0.63); or between presence of LGE and RVol (*P* = 0.17), including in the total population or in subgroups of men or women.

### Sex differences in myocardial extracellular volume and symptoms

In the total population, the median ECV fraction was higher in women compared to men (26.4 ± 44.5% vs. 25.2 ± 44.6% *P* = 0.03). Diabetes was positively associated with increased ECV (*P* = 0.03) and no significant association between ECV and patients’ age, aortic valve leaflet morphology, hypertension, hyperlipidemia, symptoms, LV volumes, or LVEF (all *P* ≥ 0.1).

In a group of normal volunteers scanned at our institution (n = 28), no statistically significant difference in ECV was noted in between male (median ECV 24 ± 4%) and females (median ECV 25 ± 4%) (*P* = 0.29). No statistically significant difference in ECV was seen between 1.5 Tesla and 3 Tesla scans (*P* = 0.42).

**Figure 3:**
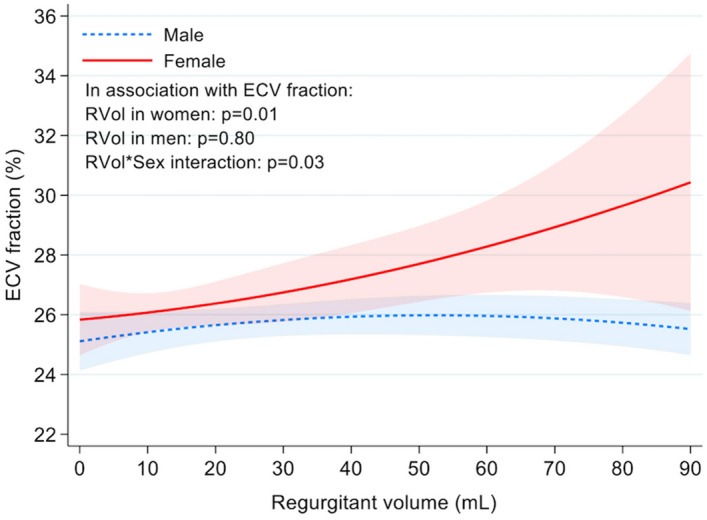
Sex differences in ECV according to AR severity. Data truncated for regurgitant volume at 90 ml. The shaded areas reflect the 95% confidence intervals. *ECV* extracellular volume fraction.

**Figure 4:**
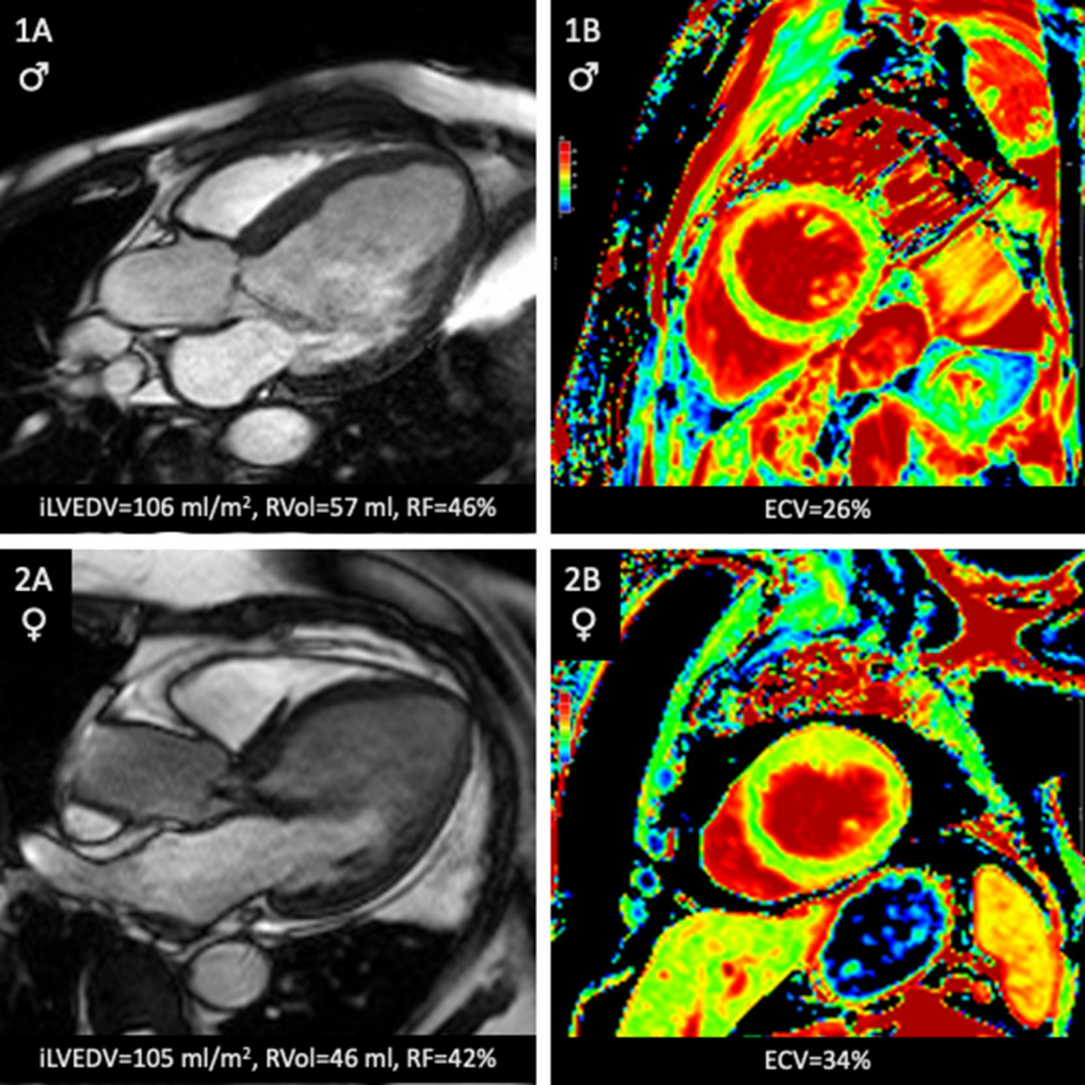
Patient examples: Panels 1A and 1B are from a 72-year-old man with moderate to severe AR. Panels 2A and 2B are from a 79-year-old woman with moderate-severe AR and elevated ECV. *ECV* extracellular volume fraction, *Ilvedv* indexed left ventricular end diastolic volume, *RVol* regurgitant volume, *RF* regurgitant fraction.

In the multivariable generalized linear model, there was a sex and RVol interaction that was independently associated with ECV, despite adjustment for leaflet morphology, RVol, and diabetes (*P* = 0.03). In women, increased RVol was positively associated with increased ECV, but not in men (Table 3 and Fig. 3). Figure 4 shows patient examples. The use of cardiac medications such as beta blockers or Renin Angiotensin Aldosterone system inhibitors was not associated with ECV (supplemental Table 1).

**Table 3:** Multivariable analysis of factors associated with ECV.

Characteristic	All patients		Female		Male	
	Adjusted β coef	*P*-value	Adjusted β coef	*P*-value	Adjusted β coef	*P*-value
	(95% CI)		(95% CI)		(95% CI)	
Female vs male	0.03 (− 1.10, 1.17)	0.95	–	–	–	–
Diabetes	1.37 (− 0.08, 2.81)	0.06	2.56 (0.20, 4.93)	0.03	0.65 (− 1.18, 2.49)	0.48
Aortic RVol	0.04 (0.00, 0.07)	0.03	0.03 (0.00, 0.07)	0.04	0.00 (− 0.02, 0.01)	0.81
Valve morphology	− 0.01 (− 0.77, 0.75)	0.98	0.34 (− 0.94, 1.62)	0.61	− 0.23 (− 1.18, 0.72)	0.64
Gender * RVol interaction	− 0.04 (− 0.08, 0.00)	0.03	–	–	–	–

In ≥ moderate AR, women were more likely to have symptoms (31.6% with ≥ NYHA class II compared to 11.1% of men, *P* = 0.02). Follow up on aortic valve replacement (AVR) vs medical management was available in 98 of 100 patients with ≥ moderate AR (median follow up duration 35.2 [14.2, 57.5] months). Sixty patients (61.2%) underwent AVR at a median of 27 [10, 53] days from the CMR study date; and 38 (38.8%) were managed medically at the end of follow up. However, the rate of AVR was similar between men and women (55.6% of women vs. 62.5% of men), *P* = 0.58.

## Discussion

The main findings of this study are: Compared to men, women had smaller ventricular volumes and mass across the spectrum of aortic regurgitation severity. These differences were not fully corrected when indexing ventricular parameters to body surface area. Our study is the first to evaluate sex differences in tissue characteristics in AR. We found that women manifested an increase in ECV at higher degrees of regurgitant volume, whereas men did not. In contrast, myocardial replacement fibrosis as assessed by LGE is relatively uncommon in isolated AR and is not associated with sex or AR severity. Women with AR were more likely to have NYHA class II or greater symptoms than men but were not more likely to undergo surgery than men.

### Sex differences in myocardial remodeling

Sex differences in myocardial remodeling to VHD have been a focus of significant research, predominantly in aortic stenosis and mitral regurgitation. However, severe AR and bicuspid aortic valve, one of its leading causes, is more common in men. As a result, there were few women in the studies that established treatment guidelines for AR^4,5^. Indeed, Klodas et al. later demonstrated worse 10-year survival in women who underwent aortic valve surgery for severe AR, potentially due to delayed referral for surgery in women^25^. The use of indexed LV end systolic diameter cutoff of 2.5 cm/m^2^ or less as a trigger for surgery can potentially lead to a timelier referral for surgery^26,27^. A more contemporary AR cohort had similar survival between men and women after aortic valve replacement^26^.

A few studies directly compared AR related myocardial remodeling in men and women. An echocardiographic study by Rohde et al. included 33 patients with isolated severe AR, 9 of which were women. Women with isolated AR had smaller indexed LV mass, LVEDV and LVESV, despite a similar degree of AR^28^. More recently, a CMR and echocardiographic study by Tower-Rader et al.^14^ found that women had smaller indexed LVEDV and LVESV compared to men particularly at higher degrees of AR, with underestimation of ventricular dimensions by echocardiography in women at higher degrees of AR.

Despite indexing by body surface area, LV volumes and mass have been demonstrated to be lower in women compared with men when assessed by CMR in the Multi-Ethnic Study of Atherosclerosis (MESA) cohort^29^. This was also seen in our study at lower degrees of regurgitant volume. However, as opposed to MESA and Tower Rader et al., we found overall similar indexed LVEDV and LV mass between men and women at regurgitant volume > 30 ml. This could suggest that women incur more LV remodeling than men for the same degree of significant AR but could still be related to relatively lower number of women with advanced degrees of AR. Larger multicenter studies are needed to evaluate this further, and investigating post-operative outcomes in women compared to men might delineate whether this would translate in differences in heart failure incidence, as was demonstrated in mitral regurgitation^13^.

### Sex Differences in myocardial tissue characteristics

It has been long recognized that the pressure and volume overload exerted on the left ventricle by AR results in cellular and extracellular changes, including myocardial fibrosis^7,30–35^. Myocardial fibrosis in AR is thought to be related to increased fibronectin and glucosamine expression with altered collagen expression and organization^8,34^. To the best of our knowledge, no prior studies have examined sex differences in the extent of myocardial fibrosis.

The development of tissue characterization techniques by CMR has allowed for the non-invasive assessment of cardiac fibrosis and proved to be prognostically important in aortic stenosis and other disease states. A small CMR study by Sparrow et al. demonstrated that myocardial T1 mapping has the potential for showing differences between relaxation times in AR and in normal hearts^36^. In another study that included 9 patients with severe AR, ECV measured on 3 Tesla CMR was strongly correlated with the extent of interstitial fibrosis on histology (r = 0.79, *P* = 0.011)^9^. There was no significant relationship between the amount of LGE and the magnitude of fibrosis determined by histology. Conversely, in a study that included 26 severe AR patients, LGE was present in 69% of patients and the correlation between LGE and histology was good (r = 0.70, *P* < 0.001). ECV was not assessed^37^.

It is unclear why women develop an increase in ECV with higher regurgitant volume. It is possible that AR is associated with activity of the renin–angiotensin–aldosterone system, which is implicated in cardiac fibrosis and has known sex differences in its activation in animal models of AR^38^. The extent of cellular versus extracellular remodeling is another potential explanation. Women may have a greater degree of extracellular remodeling than men, leading to a higher ECV with increasing regurgitation severity, while men do not. Similar to LV cavity remodeling, this finding could also suggest that women are manifesting relatively greater degrees of interstitial fibrosis than men for the same degree of significant AR; and that AR severity thresholds may be different in men and women.

## Clinical implications

Our investigation highlights important differences and similarities between men and women with AR. Indexing LV volumes and mass to BSA appears to correct for the differences between men and women across patients with ≥ moderate AR. But as discussed above, indexed LV volumes and mass by CMR were larger in men than in women in MESA, suggesting women may be incurring greater increases in LV volume and mass than men do for the same degree of AR. Our study also highlights the importance of indexing LV parameters. Although indexing LV end systolic diameter to body surface area is recommended by the guidelines particularly for women and small patients^39^, the recommendation is less likely to be uniformly followed across practices compared to thresholds of symptoms, LVEF and absolute LV end systolic diameter, which may lead to an unnecessary delay of treatment in women. This is supported by the finding of women being more likely to have symptoms than men at ≥ moderate AR but undergoing surgery at a similar rate. However, confirmation of these findings is needed considering the relatively small number of women with severe AR in our study.

The observation of increased ECV in women with increasing regurgitant volume needs confirmation in larger independent cohorts; in addition to longitudinal follow up to determine if an increase in ECV translates to differences in symptoms, reverse remodeling after surgery, and long-term outcomes.

## Limitations

The spectrum of AR severity was different between men and women, and the number of women, particularly those with severe AR was small as three out of four patients with severe AR are men^26^. In addition, a potential selection bias in referring patients to CMR can not be excluded in our study. This may also have influenced the findings on ejection fraction and LVESV and their differences in men versus women, if the rates and timing of referral to CMR is different between men and women. We attempted to alleviate this bias by using generalized linear modeling analysis of continuous variables and controlling for confounders in ECV analysis, particularly leaflet morphology (bicuspid vs trileaflet valves) and diabetes status. Despite this limitation, we believe it is imperative to study AR in women considering their relative under-representation in previous and current research studies of AR.

Not all patients enrolled underwent specific testing to rule out other possible confounding causes of myocardial fibrosis (i.e., coronary angiography). However, clinical history and available diagnostic testing results were thoroughly evaluated to minimize confounding factors. ECV fraction is not specific to fibrosis and is slightly higher in women compared to men in healthy adults. However, multiple studies have validated ECV against myocardial fibrosis in histologic specimens including in VHD. ECV is also positively associated with diabetes, but we controlled for the presence of diabetes in multivariable analysis and its prevalence was not different between men and women. The CMR results may influence the decision to proceed with surgery, and conclusions about the rate of surgery in men vs women should be tempered by this potential bias. Finally, we present our results according to regurgitant volume, not fraction. It could be argued that for the purposes of LV remodeling assessment, the regurgitant volume might be a better representative marker of the hemodynamic load (not necessarily outcomes); since the regurgitant fraction corrects for the smaller stroke volume in women and might obscure some sex differences in these parameters.

## Conclusions

Our study demonstrates sex differences in ventricular cavity and tissue remodeling in aortic regurgitation. Indexing ventricular parameters to body surface area does not fully resolve differences ventricular volumes between men and women, Women demonstrated an increase in ECV at higher degrees of regurgitant volume, whereas men do not. In contrast, myocardial replacement fibrosis as assessed by LGE is relatively uncommon in isolated AR and is not associated with sex or AR severity. Women were more likely to have symptoms than men at ≥ moderate AR but underwent surgery at a similar rate. Further research is required to confirm these findings and investigate their implications on development of symptoms, determining the optimal timing of surgery, reverse remodeling after surgery, and long-term outcomes.

## Supplementary Information

Supplementary Table 1.

## Data Availability

The datasets generated and/or analyzed during the current study are not publicly available due Houston Methodist Research Institute policies but are available from the corresponding author on reasonable request and with permission of the Houston Methodist Research Institute.

## References

[1] Wisenbaugh T, Spann JF, Carabello BA (1984). Differences in myocardial performance and load between patients with similar amounts of chronic aortic versus chronic mitral regurgitation. J. Am. Coll. Cardiol..

[2] Singh JP, Evans JC, Levy D, Larson MG, Freed LA, Fuller DL, Lehman B, Benjamin EJ (1999). Prevalence and clinical determinants of mitral, tricuspid, and aortic regurgitation (the Framingham Heart Study). Am. J. Cardiol..

[3] Carabello BA (1990). Aortic regurgitation. A lesion with similarities to both aortic stenosis and mitral regurgitation. Circulation.

[4] Bonow RO, Dodd JT, Maron BJ, O’Gara PT, White GG, McIntosh CL, Clark RE, Epstein SE (1988). Long-term serial changes in left ventricular fuction and reversal of ventricular dilatation after valve replacement for chronic aortic regurgitation. Circulation.

[5] Bonow RO, Lakatos E, Maron BJ, Epstein SE (1991). Serial long-term assessment of the natural history of asymptomatic patients with chronic aortic regurgitation and normal left ventricular systolic function. Circulation.

[6] Dujardin KS, Enriquez-Sarano M, Schaff HV, Bailey KR, Seward JB, Tajik AJ (1999). Mortality and morbidity of aortic regurgitation in clinical practice. A long-term follow-up study. Circulation.

[7] Borer JS, Herrold EMM, Carter JN, Catanzaro DF, Supino PG (2006). Cellular and molecular basis of remodeling in valvular heart diseases. Heart Fail. Clin..

[8] Borer JS, Truter S, Herrold EM, Falcone DJ, Pena M, Carter JN, Dumlao TF, Lee JA, Supino PG (2002). Myocardial fibrosis in chronic aortic regurgitation molecular and cellular responses to volume overload. Circulation.

[9] De Meester De Ravenstein C, Bouzin C, Lazam S, Boulif J, Amzulescu M, Melchior J, Pasquet A, Vancraeynest D, Pouleur AC, Vanoverschelde JLJ, Gerber BL (2015). Histological validation of measurement of diffuse interstitial myocardial fibrosis by myocardial extravascular volume fraction from modified look-locker imaging (MOLLI) T1 mapping at 3 T. J. Cardiovasc. Magn. Reson..

[10] Treibel TA, Kozor R, Fontana M, Torlasco C, Reant P, Badiani S, Espinoza M, Yap J, Diez J, Hughes AD, Lloyd G, Moon JC (2018). Sex dimorphism in the myocardial response to aortic stenosis. JACC Cardiovasc. Imaging.

[11] Tastet L, Kwiecinski J, Pibarot P, Capoulade R, Everett RJ, Newby DE, Shen M, Guzzetti E, Arsenault M, Bédard É, Larose É, Beaudoin J, Dweck M, Clavel MA (2019). Sex-related differences in the extent of myocardial fibrosis in patients with aortic valve stenosis. JACC Cardiovasc. Imaging.

[12] Singh A, Chan DCS, Greenwood JP, Dawson DK, Sonecki P, Hogrefe K, Kelly DJ, Dhakshinamurthy V, Lang CC, Khoo JP, Sprigings D, Steeds RP, Zhang R, Ford I, Jerosch-Herold M, Yang J, Li Z, Ng LL, McCann GP (2019). Symptom onset in aortic stenosis. JACC Cardiovasc. Imaging.

[13] Mantovani F, Clavel MA, Michelena HI, Suri RM, Schaff HV, Enriquez-Sarano M (2016). Comprehensive imaging in women with organic mitral regurgitation implications for clinical outcome. JACC Cardiovasc. Imaging.

[14] Tower-Rader A, Mathias IS, Obuchowski NA, Kocyigit D, Kumar Y, Donnellan E, Bolen M, Phelan D, Flamm S, Griffin B, Cho L, Svensson LG, Pettersson G, Popovic Z, Kwon D (2022). Sex-based differences in left ventricular remodeling in patients with chronic aortic regurgitation: A multi-modality study. J. Cardiovasc. Magn. Reson..

[15] Senapati A, Malahfji M, Debs D, Yang EY, Nguyen DT, Graviss EA, Shah DJ (2021). Regional replacement and diffuse interstitial fibrosis in aortic regurgitation: Prognostic implications from cardiac magnetic resonance. JACC Cardiovasc. Imaging.

[16] Malahfji M, Senapati A, Tayal B, Nguyen DT, Graviss EA, Nagueh SF, Reardon MJ, Quinones M, Zoghbi WA, Shah DJ (2020). Myocardial scar and mortality in chronic aortic regurgitation. J. Am. Heart Assoc..

[17] Kitkungvan D, Nabi F, Kim RJ, Bonow RO, Khan MA, Xu J, Little SH, Quinones MA, Lawrie GM, Zoghbi WA, Shah DJ (2018). Myocardial fibrosis in patients with primary mitral regurgitation with and without prolapse. J. Am. Coll. Cardiol..

[18] Kitkungvan D, Yang EY, el Tallawi KC, Nagueh SF, Nabi F, Khan MA, Nguyen DT, Graviss EA, Lawrie GM, Zoghbi WA, Bonow RO, Quinones MA, Shah DJ (2021). Extracellular volume in primary mitral regurgitation. JACC Cardiovasc. Imaging.

[19] Kitkungvan D, Yang EY, el Tallawi KC, Nagueh SF, Nabi F, Khan MA, Nguyen DT, Graviss EA, Lawrie GM, Zoghbi WA, Bonow RO, Quinones MA, Shah DJ (2019). Prognostic implications of diffuse interstitial fibrosis in asymptomatic primary mitral regurgitation. Circulation.

[20] Yang EY, Ghosn MG, Khan MA, Gramze NL, Brunner G, Nabi F, Nambi V, Nagueh SF, Nguyen DT, Graviss EA, Schelbert EB, Ballantyne CM, Zoghbi WA, Shah DJ (2019). Myocardial extracellular volume fraction adds prognostic information beyond myocardial replacement fibrosis. Circ. Cardiovasc. Imaging.

[21] Kawel-Boehm N, Maceira A, Valsangiacomo-Buechel ER, Vogel-Claussen J, Turkbey EB, Williams R, Plein S, Tee M, Eng J, Bluemke DA (2015). Normal values for cardiovascular magnetic resonance in adults and children. J. Cardiovasc. Magn. Reson..

[22] Schulz-Menger J, Bluemke DA, Bremerich J, Flamm SD, Fogel MA, Friedrich MG, Kim RJ, Von Knobelsdorff-Brenkenhoff F, Kramer CM, Pennell DJ, Plein S, Nagel E (2013). Standardized image interpretation and post processing in cardiovascular magnetic resonance: Society for cardiovascular magnetic resonance (SCMR) board of trustees task force on standardized post processing. J. Cardiovasc. Magn. Reson..

[23] Debs D, Senapati A, Malahfji M, Yang E, Shah D (2019). Regional replacement and diffuse interstitial left ventricular fibrosis in chronic aortic valve regurgitation assessed by cardiac magnetic resonance. J. Am. Coll. Cardiol..

[24] Tayal B, Debs D, Nabi F, Malahfji M, Little SH, Reardon M, Zoghbi W, Kleiman N, Shah DJ (2020). Impact of myocardial scar on prognostic implication of secondary mitral regurgitation in heart failure. JACC Cardiovasc. Imaging.

[25] Klodas E, Enriquez-Sarano M, Tajik AJ, Mullany CJ, Bailey KR, Seward JB (1996). Surgery for aortic regurgitation in women. Circulation.

[26] Mentias A, Feng K, Alashi A, Rodriguez LL, Gillinov AM, Johnston DR, Sabik JF, Svensson LG, Grimm RA, Griffin BP, Desai MY (2016). Long-term outcomes in patients with aortic regurgitation and preserved left ventricular ejection fraction. J. Am. Coll. Cardiol..

[27] Yang LT, Michelena HI, Scott CG, Enriquez-Sarano M, Pislaru SV, Schaff HV, Pellikka PA (2019). Outcomes in chronic hemodynamically significant aortic regurgitation and limitations of current guidelines. J. Am. Coll. Cardiol..

[28] Rohde LEP, Zhi G, Aranki SF, Beckel NE, Lee RT, Reimold SC (1997). Gender-associated differences in left ventricular geometry in patients with aortic valve disease and effect of distinct overload subsets. Am. J. Cardiol..

[29] Liu CY, Liu YC, Wu C, Armstrong A, Volpe GJ, Van Der Geest RJ, Liu Y, Hundley WG, Gomes AS, Liu S, Nacif M, Bluemke DA, Lima JAC (2013). Evaluation of age-related interstitial myocardial fibrosis with cardiac magnetic resonance contrast-enhanced T1 mapping: MESA (Multi-Ethnic Study of Atherosclerosis). J. Am. Coll. Cardiol..

[30] Fuster V, Danielson MA, Robb RA, Broadbent JC, Brown AL, Elveback LR (1977). Quantitation of left ventricular myocardial fiber hypertrophy and interstitial tissue in human hearts with chronically increased volume and pressure overload. Circulation.

[31] Maron BJ, Ferrans VJ, Roberts WC (1975). Myocardial ultrastructure in patients with chronic aortic valve disease. Am. J. Cardiol..

[32] Schwarz F, Flameng W, Schaper J, Langebartels F, Sesto M, Hehrlein F, Schlepper M (1978). Myocardial structure and function in patients with aortic valve disease and their relation to postoperative results. Am. J. Cardiol..

[33] Krayenbuehl HP, Hess OM, Monrad ES, Schneider J, Mall G, Turina M (1989). Left ventricular myocardial structure in aortic valve disease before, intermediate, and late after aortic valve replacement. Circulation.

[34] Piper C, Schultheiss HP, Akdemir D, Rudolf J, Horstkotte D, Pauschinger M (2003). Remodeling of the cardiac extracellular matrix differs between volume- and pressure-overloaded ventricles and is specific for each heart valve lesion. J. Heart Valve Dis..

[35] Liu SK, Magid NR, Fox PR, Goldfine SM, Borer JS (1998). Fibrosis, myocyte degeneration and heart failure in chronic experimental aortic regurgitation. Cardiology.

[36] Sparrow P, Messroghli DR, Reid S, Ridgway JP, Bainbridge G, Sivananthan MU (2006). Myocardial T1 mapping for detection of left ventricular myocardial fibrosis in chronic aortic regurgitation: Pilot study. Am. J. Roentgenol..

[37] Azevedo CF, Nigri M, Higuchi ML, Pomerantzeff PM, Spina GS, Sampaio RO, Tarasoutchi F, Grinberg M, Rochitte CE (2010). Prognostic significance of myocardial fibrosis quantification by histopathology and magnetic resonance imaging in patients with severe aortic valve disease. J. Am. Coll. Cardiol..

[38] Walsh-Wilkinson É, Drolet MC, Le Houillier C, Roy ÈM, Arsenault M, Couet J (2019). Sex differences in the response to angiotensin II receptor blockade in a rat model of eccentric cardiac hypertrophy. PeerJ.

[39] Nishimura, R. A., Otto, C. M., Bonow, R. O., Carabello, B. A., Erwin, J. P, Guyton, R. A., O’Gara, P. T., Ruiz, C. E., Skubas, N. J., Sorajja, P., Sundt, T. M. & Thomas, J. D. 2014 AHA/ACC guideline for the management of patients with valvular heart disease: A report of the American college of cardiology/American heart association task force on practice guidelines. *J. Am. Coll. Cardiol*. **63 **(22) (2014). 10.1016/j.jacc.2014.02.536 24603191

