# Supplementary material for: Sex differences in myocardial remodeling and extracellular volume in aortic regurgitation
Source: Sci Rep. 2023 Jul 13;13:11334. doi: 10.1038/s41598-023-37444-y (PMC10344872; doi:10.1038/s41598-023-37444-y)
Supplement: Supplementary file 1 — Supplementary Table 1. [file 41598_2023_37444_MOESM1_ESM.docx]

| **Supplemental Table 1. Univariable generalized linear model (GLM) for factors associated with ECV** | | |
| --- | --- | --- |
| **Characteristic** | **Unadjusted  β coef.** | **p-value** |
|  | **(95% CI)** |  |
| **Medications** |  |  |
| Beta blockers | 0.16 (-0.60, 0.91) | 0.69 |
| ACE inhibitor | 0.18 (-0.70, 1.05) | 0.70 |
| ARB | -0.34 (-1.25, 0.57) | 0.46 |
| Spironolactone | -1.47 (-3.81, 0.88) | 0.22 |
| Diuretics | 0.65 (-0.23, 1.52) | 0.15 |
